# Autoimmune Thyroiditis in Childhood

**DOI:** 10.4274/Jcrpe.855

**Published:** 2013-03-01

**Authors:** Rosalind S. Brown

**Affiliations:** 1 Division of Endocrinology, Children’s Hospital Boston and Department of Pediatrics, Harvard Medical School, Boston, USA

**Keywords:** Thyroiditis, hypothyroidism, children, Autoimmunity

## Abstract

Autoimmune thyroiditis (AIT) is the most common thyroid disorder in the pediatric age range. The disease results from an as yet poorly characterized defect or defects in immunoregulation and a cascade of events progressing from lymphocyte infiltration of the thyroid, to T-cell- and cytokine-mediated thyroid follicular cell injury, and apoptotic cell death. Approximately 70% of disease risk has been attributed to genetic background with environmental factors being important in triggering disease in susceptible individuals. The contribution of individual genes is small and probably polymorphisms in multiple genes play a role. Some immunosusceptibility genes affect immune recognition or response in general, while others are thyroid-specific. Environmental agents may act through an epigenetic mechanism. Antibodies (Abs) to a variety of thyroid-specific antigens are detectable in a majority of patients, but the role of Abs in mediating cell injury and death is unclear and only thyrotropin (TSH) receptor Abs significantly affect thyroid function by interfering with (or stimulating) the action of TSH. Nonetheless, thyroid peroxidase (TPO) Abs and thyroglobulin (Tg) Abs, present in a majority of patients, are valuable diagnostically as markers of underlying autoimmune thyroid destruction. TSH receptor blocking Abs are found in ˜18% of children and adolescents with severe hypothyroidism and, when persistent, may identify an adolescent likely to have a baby with TSH receptor blocking Ab-induced congenital hypothyroidism. AIT may coexist with other organ-specific autoimmune diseases. Although the most common age at presentation is adolescence, the disease may occur rarely in children <1 year of life.

**Conflict of interest:**None declared.

## INTRODUCTION

Autoimmune thyroiditis (AIT) is the most common thyroid disorder in the pediatric age range. Both a goitrous (Hashimoto’s thyroiditis) and a nongoitrous (atrophic thyroiditis, also called primary myxedema) variant of AIT have been distinguished. In pediatrics, the most common age at presentation is adolescence, but the disease may occur at any time, rarely even in children under one year of age (1). In the past decade, the application of molecular biology has permitted an unparalleled insight into susceptibility genes that predispose to the development of AIT and into its complex immune pathophysiology. Approximately 70% of disease risk is related to genetic susceptibility with environmental factors playing a role in triggering disease in susceptible individuals. In this brief review, current concepts about the immuno-pathogenesis and molecular genetics of AIT as well as putative environmental triggers will be discussed. In addition, clinical aspects unique to the pediatric age range will be reviewed. 

**Immuno-pathogenesis of Autoimmune Thyroiditis Cellular İmmune Responses**

Because of the importance of T- cells in immune regulation, much attention has focused on this lymphocyte subpopulation to explain the breakdown in tolerance and the clinical manifestations seen in autoimmune thyroid disease (AITD). Consistent with their playing a fundamental role, an increased proportion of activated T-helper (Th) (CD4+) cells can be demonstrated in the circulation of a majority of patients with AIT (2) and this is thought to lead to a cascade of immune-mediated events. Lymphocytic infiltration of the thyroid gland and organization into lymphoid follicles occur due to the secretion of numerous chemokines that direct leukocyte migration and to adhesion molecules that lead to cell attachment to extracellular matrix proteins. These processes are accompanied by the secretion of complement, cytokines and other soluble mediators that damage the thyrocyte and lead ultimately to cell death by apoptosis (3). It is of interest that not only ‘professional’ antigen-presenting cells (e.g., macrophages, dendritic cells) but thyroid follicular cells themselves play a crucial role in determining disease progression by expressing major histocompatibility complex (MHC) class II antigens (4) and secreting cytokines and chemokines. Goiter, present in approximately two-thirds of children with AIT, results from both cellular infiltration and thyroid follicular cell proliferation, a consequence of the compensatory increase in thyrotropin (TSH) that occurs in response to thyrocyte dysfunction. 

To add further to the complexity, there are actually 2 types of Th cells: Th1 and Th2, each with a different pattern of cytokine production, effector function, chemokine receptors, and regulation (5). Th1 cells subserve cell-mediated immune responses, while Th2 cells are involved in antibody production. It has been postulated that in AIT, a defect in a specific T cell subpopulation, termed regulatory T cells (Tregs), results in some way in a change in the thyroid microenvironment, leading to decreased inhibition of Th1 cells and the overproduction of Th1 cytokines. 

**Humoral İmmunity**

In addition to cell-mediated immune mechanisms, AIT is characterized by the secretion of antibodies (Abs) to a variety of thyroid-specific antigens, most notably thyroglobulin (Tg), and thyroid peroxidase (TPO) but also to a lesser extent the TSH receptor, the sodium iodide symporter (NIS) (6) and most recently pendrin (7). Whether, and, if so, how antibody-mediated immune mechanisms contribute to the initiation, progression or pathogenesis of AIT remains unclear. Nonetheless, measurement of Abs to Tg and TPO is useful diagnostically as markers of underlying autoimmunity, and TSH receptor Abs may modulate the activity of the TSH receptor, thereby affecting thyroid function in a subset of patients. The role of Abs to NIS or pendrin is not yet clear.

**Antibodies to Thyroglobulin and Thyroid Peroxidase**

Tg Abs are directed at Tg, the very large 660 kDA homodimeric protein that serves as the storage form and precursor of thyroid hormone. There is some evidence that Tg Abs in patients with AIT are more restricted in their epitope specificity than in healthy individuals, but the antigenic determinants recognized by Tg Abs are not known (8). Post-translational modifications such as iodination and glycosylation may play a role in Tg antigenicity (9). TPO Abs recognize TPO, the key membrane-associated enzyme on the apical surface of the thyrocyte that mediates the iodination and coupling of iodotyrosines to form thyroid hormone (10). Both cytotoxicity (e.g., activation of the complement cascade and participation in Ab-dependent, cell-mediated cytotoxicity ) and TPO Ab-mediated inhibition of TPO function have been reported in vitro, but these effects do not appear to be important in vivo as indicated by the failure of these Abs to affect fetal or neonatal thyroid function when transmitted to the fetus. Like with Tg Abs, some epitopic specificity of TPO Abs has been reported in patients with AIT (11), the significance of which is unclear.

**TSH Receptor Antibodies**

Abs to the TSH receptor have been classified as stimulatory, blocking and neutral Abs (12). All varieties recognize specific linear epitopes presented in a 3-dimensional conformation , but their interaction differs subtly. Like other members of the G-coupled receptor superfamily, the TSH receptor is composed of a large, N-terminal ectodomain, 7-transmembrane-spanning regions and a small intracytoplasmic tail. Although initial studies suggested separate epitopes for stimulatory Abs on the N-terminus and for blocking Abs on the C-terminus of the ectodomain, recent evidence suggests more overlap than previously thought (12). Stimulatory Abs appear to be of limited heterogeneity, whereas blocking Abs are not similarly restricted. 

Both blocking and stimulating TSH receptor Abs can be found in some patients with AIT. The coexistence of stimulatory TSH receptor Abs and AIT has been termed ‘Hashitoxicosis’. In contrast, blocking Abs may contribute to the severity of the hypothyroidism by inhibiting TSH-induced cell proliferation and hormonogenesis. In a recent study of children and adolescents, TSH receptor blocking Abs were found in 17.8% of patients with severe hypothyroidism (defined as a serum TSH concentration >20 mU/L). However, unlike in adults, they were found in goitrous as well as nongoitrous patients and, when present in high concentrations, appeared to persist indefinitely (13). These data suggest that the presence of potent TSH receptor blocking Abs in adolescent females may identify patients at risk of having babies with transient congenital hypothyroidism in their future ages. 

**Immunosusceptibility Genes**

Like Graves disease (GD) with which it is closely associated, AIT is a complex immune disorder that occurs in individuals with an underlying genetic susceptibility. As many as 20-60 immunosusceptibility genes, each with small effect, have been postulated (14). Some genes are involved in immune recognition and/or response in general, while others are thyroid-specific; certain genes are common to both AIT and GD, while others tend to predominate only in GD (8,9). In this brief review, only those that affect individuals with AIT will be discussed. These genes are summarized in Table 1.

**Immune Response Genes**

Human leukocyte antigen (HLA): The MHC region encodes a highly polymorphic region that includes the genes for the class I (A, B and C) and class II (DR, DP and DQ) HLA. As peptide antigens are presented to T cells only when bound to HLA class II molecules, the HLA haplotype plays a critical role in determining which antigens will be recognized by the T-cell receptor and trigger an immune response. Unlike with GD, no consistent HLA-association has been identified in patients with AIT. Recently, Menconi et al (15) sequenced the polymorphic exon 2 of the HLA-DR gene in 94 patients with AIT and 149 controls. They identified a four-amino acid haplotype (Tyr-26, Tyr-30, Gln-70, Lys-71) that conferred an odds ratio of 3.73 irrespective of the HLA haplotype; the single amino acid Lys-71 conferred an odds ratio of 2.98. These investigators postulated that specific ‘pocket amino acid signatures’ determine susceptibility to AIT by causing critical structural changes that influence antigenic peptide binding and presentation.

**Cytotoxic T Lymphocyte Antigen-4**

Cytotoxic T lymphocyte (CTLA)-4, a transmembrane protein of the immunoglobulin superfamily, is an important costimulatory molecule that downregulates T-cell activation by binding to the B7 molecules present on antigen-presenting cells and preventing the second signal necessary for T-cell activation. Linkage of the CTLA-4 region with thyroid autoantibody production in patients with and without clinical thyroid disease has been demonstrated (16). This suggests that additional factors must be required for the development of a thyroid functional abnormality.

**Protein Tyrosine Phosphatase-22**

Protein tyrosine phosphatase 22 (PTPN22) encodes a protein, lymphoid tyrosine phosphatase (LYP), which is a potent inhibitor of the T-cell receptor signaling pathway . A tryptophan for arginine substitution at codon 620 of the LYP molecule has been associated with both GD and AIT in some but not all ethnic populations (16).

**Thyroid-specific Gene**

The thyroid-specific genes that have been incriminated in AITD include those that code for Tg and the TSH receptor, but only the Tg gene has been associated with AIT.

**Thyroglobulin**

Tg is a very large 660kDA homodimeric protein that functions as the storage form and precursor of thyroid hormone. In genetically susceptible mice, experimental AIT can be induced by Tg immunization and there is evidence that Tg sensitization may play a role in patients with AITD as well. Detailed sequence analysis of the Tg gene has demonstrated significant associations between specific polymorphisms of Tg SNPs and AIT (**17**,**18**). These findings suggest that specific Tg polymorphisms might be more optimally presented to T cells by specific HLA haplotypes, thereby triggering an immune response. This is highly reminiscent of findings in experimental animals in which induction of AIT is also HLA-dependent. 

In addition, the immunogenicity of Tg has been related to the extent of its iodination (**19**). The pathogenic mechanisms whereby increased iodine predisposes to AIT remain unclear.

**Environmental Factors**

The precise environmental trigger(s) leading to the development of disease is not known with certainty, but infection, drugs (lithium, amiodarone, interferon-alpha), hormones (estrogen), dietary substances (iodine, selenium), stress, smoking and, most recently, environmental toxins have all been implicated (20,21). An epigenetic mechanism has been postulated in some cases (21). 

**Incidence and Associations**

AIT is more common in North America and Japan than in some iodine-deficient parts of Europe and there is a striking female preponderance, even prior to puberty. AIT may occur alone or may coexist with other organ-specific autoimmune diseases, particularly type 1 diabetes mellitus (T1DM). The combination of AIT with specific autoimmune disorders has been termed the autoimmune polyglandular syndromes (APS). APS 1, which tends to present in the first decade of life and is caused by a mutation in a single (‘autoimmune regulator’) gene, is characterized by the sequential development of mucocutaneous candidiasis, hypoparathyroidism and adrenal deficiency (22). AIT is amongst a multiplicity of other organ-specific diseases that can also develop in a minority of affected patients. In APS-2 which tends to present later in childhood or adolescence, Addison’s disease is found in association with AIT (Schmidt syndrome) or AIT plus T1DM (Carpenter syndrome) (23). Multiple other organ-specific autoimmune diseases may also be present. AIT has also been described in children with immunodysregulation polyendocrinopathy enteropathy X-linked (IPEX) syndrome, a polyglandular disorder characterized by early-onset diabetes and colitis (24) . There is an increased incidence of AIT in patients with certain chromosomal abnormalities [Down syndrome (25), Turner syndrome (26), and, to a lesser extent, Klinefelter syndrome] as well as in patients with Noonan syndrome. AIT may be associated with chronic urticaria (27) and rarely with immune-complex glomerulonephritis (28). 

**Clinical Manifestations**

damPatients with AIT may be euthyroid, or they may have subclinical or overt hypothyroidism depending on the severity of the immunologic damage. Unexplained poor linear growth is a classical initial finding in many hypothyroid children. In some patients, an asymptomatic goiter may be noted on routine examination. Occasionally, an initial thyrotoxic phase occurs due to the discharge of preformed thyroid hormone from the aged gland. In this case, the picture needs to be distinguished from hyperthyroidism due to GD. Rarely, as noted previously, both diseases may coexist in the same patient (Hashitoxicosis). 

The typical thyroid gland in AIT is diffusely enlarged and has a rubbery consistency. Although the surface is classically described as ‘pebbly’ or bosselated, occasionally asymmetric enlargement occurs that must be distinguished from thyroid neoplasia. A palpable lymph node superior to the isthmus (‘Delphian node’) may be noted.

**Laboratory Evaluation**

Measurement of the serum TSH concentration is the best initial screening test for the presence of primary hypothyroidism. If the TSH is elevated, then evaluation of the serum free thyroxine (fT4) concentration will distinguish whether the child has subclinical (normal fT4) or overt (low fT4) hypothyroidism. A diagnosis of AIT is made by the demonstration of an elevated concentration of Tg Abs and/or TPO Abs in serum. Measurement of TSH receptor blocking Abs should be considered in adolescent females with severe hypothyroidism because of the persistence of this Ab population in some patients and its association with an increased risk of having offspring with TSH receptor blocking Ab-induced congenital hypothyroidism. Imaging studies (thyroid ultrasonography and/or thyroid uptake and scan) may be performed if thyroid Ab tests are negative or if a nodule is palpable, but are rarely necessary. Occasionally, the finding of heterogeneous echogenicity on ultrasound examination has been described prior to the appearance of Abs. However, the typical picture of spotty uptake of radioactive iodine that is seen in adults is rare in children. 

**Therapy**

In patients who present with severe, longstanding hypothyroidism, slow correction with LT4 is advisable in order to minimize the potential development of unwanted side effects (deterioration in school performance, short attention span, hyperactivity, insomnia, and behavior difficulties) (29). In such patients, the replacement dose should be increased slowly over several weeks to months. Severely hypothyroid children should also be observed closely for complaints of severe headache when therapy is initiated because of the rare development of pseudotumor cerebri. In contrast, full replacement can be initiated at once without much risk of adverse consequences in children with mild hypothyroidism.

Treatment of children and adolescents with subclinical hypothyroidism (normal fT4, elevated TSH) is controversial. In adults, particularly those > 60 years of age in whom the risk of progression to overt hypothyroidism is significant, treatment has been recommended whenever the serum TSH concentration is >10 mU/L; if TSH is in the 6-10 mU/L range, treatment on a case by case basis is suggested (30). Long-term follow-up studies of children with subclinical hypothyroidism due to AIT have suggested a significant likelihood of remission. Consequently, if there is not a strong family history of hypothyroidism and the patient is not symptomatic, a reasonable option is to reassess thyroid function in 6 months. On the other hand, some initially euthyroid patients will become hypothyroid over time. Therefore, regular follow-up is necessary.

The typical replacement dose of LT4 in childhood is approximately 100 μg/m2 or 4 to 6 μg/kg for children 1 to 5 years of age, 3 to 4 μg/kg for those aged 6 to 10 years, and 2 to 3 μg/kg for those 11 years of age and older. In patients with a goiter, a somewhat higher LT4 dosage is used so as to keep the TSH in the low normal range (0.3 to 1.0 mU/L in an ultrasensitive assay), and thereby minimize its goitrogenic effect. T4 and TSH should be measured after the child has received the recommended dosage for at least 6-8 weeks. Once a euthyroid state has been achieved, patients should be monitored every 6 to 12 months. Thyroid hormone replacement is not associated with significant weight loss in overweight children, unless the hypothyroidism is severe (31). Treatment is usually continued indefinitely.

## Figures and Tables

**Table 1:**
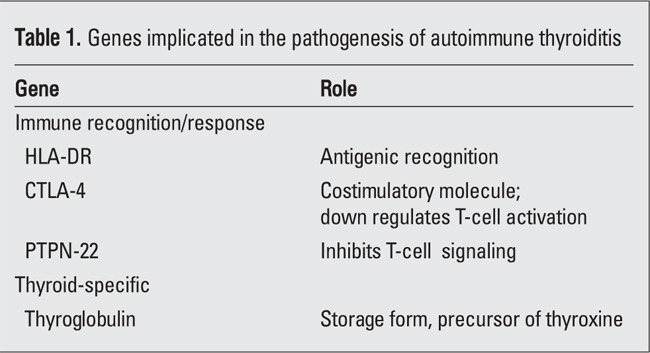
Genes implicated in the pathogenesis of autoimmune thyroiditis
